# EGFR signaling coordinates patterning with cell survival during *Drosophila* epidermal development

**DOI:** 10.1371/journal.pbio.3000027

**Published:** 2018-10-31

**Authors:** Samuel H. Crossman, Sebastian J. Streichan, Jean-Paul Vincent

**Affiliations:** 1 The Francis Crick Institute, London, United Kingdom; 2 Department of Physics, University of California, Santa Barbara, California, United States of America; Institut Pasteur, FRANCE

## Abstract

Extensive apoptosis is often seen in patterning mutants, suggesting that tissues can detect and eliminate potentially harmful mis-specified cells. Here, we show that the pattern of apoptosis in the embryonic epidermis of *Drosophila* is not a response to fate mis-specification but can instead be explained by the limiting availability of prosurvival signaling molecules released from locations determined by patterning information. In wild-type embryos, the segmentation cascade elicits the segmental production of several epidermal growth factor receptor (EGFR) ligands, including the transforming growth factor Spitz (TGFα), and the neuregulin, Vein. This leads to an undulating pattern of signaling activity, which prevents expression of the proapoptotic gene *head involution defective* (*hid*) throughout the epidermis. In segmentation mutants, where specific peaks of EGFR ligands fail to form, gaps in signaling activity appear, leading to coincident *hid* up-regulation and subsequent cell death. These data provide a mechanistic understanding of how cell survival, and thus appropriate tissue size, is made contingent on correct patterning.

## Abstract 2

Cells lacking essential patterning determinants are eliminated from developing tissues by apoptosis; this study shows that cell death in fruit fly segmentation mutants results from disruption of the pattern of EGFR signalling, which in normal embryos coordinates segment size with patterning information.

## Author summary

In many tissues, defective cells are eliminated by a process called apoptosis. This process prevents the emergence of rogue cells, which could be detrimental to normal physiology. Apoptosis is particularly apparent in developing embryos that lack appropriate positional information, and it has been suggested that in the absence of clear positional instructions, cells are unable to acquire a defined fate and commit suicide as a result. Here, we have used mutant fruit fly embryos lacking essential segmental determinants to identify the molecular signals that trigger apoptosis in response to mispatterning. We found that cells do not trigger apoptosis in response to conflicting fate determinants. Instead, mispatterning disrupts a tissue size control system that removes excess cells in oversized segments. Specifically, correct patterning information leads to the segmentally repeated production of survival signals, which activate the epidermal growth factor receptor, and this system is disrupted in patterning mutants leading to reproducible patterns of apoptosis. We propose that a similar, though less obvious, process also occurs in normal embryos. In such embryos, each segment would initially comprise a slight excess of cells and would then be trimmed down to a size specified by the pattern of survival signal production and the range of these signals. We suggest that a similar regulatory logic could ensure the coordination of tissue patterning and size in a variety of developing tissues.

## Introduction

Defective cells are often eliminated by apoptosis during development and tissue homeostasis [1–4]. This has been particularly well studied during the process of cell competition, whereby unfit cells are eliminated when confronted with normal cells within a growing tissue [5]. Excess apoptosis is also seen in mutants that lack essential developmental determinants, a phenomenon that has been observed in a variety of model organisms, including zebrafish embryos lacking the signaling molecule Sonic Hedgehog [6], mice lacking the negative Wnt signaling regulator Adenomatous polyposis coli (APC) in the developing neural crest [7], and *Drosophila* segmentation mutants [8–12]. These observations have suggested the existence of a quality control system that detects conflicting or nonsense patterning inputs and, as a result, initiates apoptosis in response.

Although apoptosis was first observed in *Drosophila* segmentation mutants over 30 years ago [8–10], relatively little is known about the molecular basis of cell elimination in this context. Previous studies have revealed that apoptosis occurs within and around the areas where the missing developmental regulators are normally required. Thus, in the pair-rule mutant *odd-skipped* (*odd*), apoptosis is seen in alternate stripes that encompass the areas where *odd* is normally expressed [13,14]. Likewise, in embryos lacking the anterior determinant Bicoid, ectopic cell death occurs primarily at the anterior of the embryonic epidermis [13]. In *Drosophila*, apoptosis is initiated by a double inhibition mechanism: the activity of ubiquitously expressed inhibitor of apoptosis proteins (IAPs), which prevent caspase activation, is inhibited by the so-called IAP antagonists *reaper* (*rpr*), *head involution defective* (*hid*), *grim*, and *sickle* (*skl*) [15]. Most apoptotic events are initiated by the transcriptional up-regulation of one or more IAP antagonists. Indeed, *hid* is up-regulated in a pattern that prefigures apoptosis in segmentation mutants, i.e., at the anterior of *bicoid* mutants and in alternate stripes in *odd* mutants [13]. Therefore, it appears that cells within the segmental pattern “know” that they are missing key fate determinants and activate *hid* expression in response. Here, we take advantage of the genetic tools in *Drosophila* to investigate how apoptosis is triggered in mispatterned epidermal cells.

## Results

### Hid mediates apoptosis in *Drosophila* segmentation mutants

**Fig 1:**
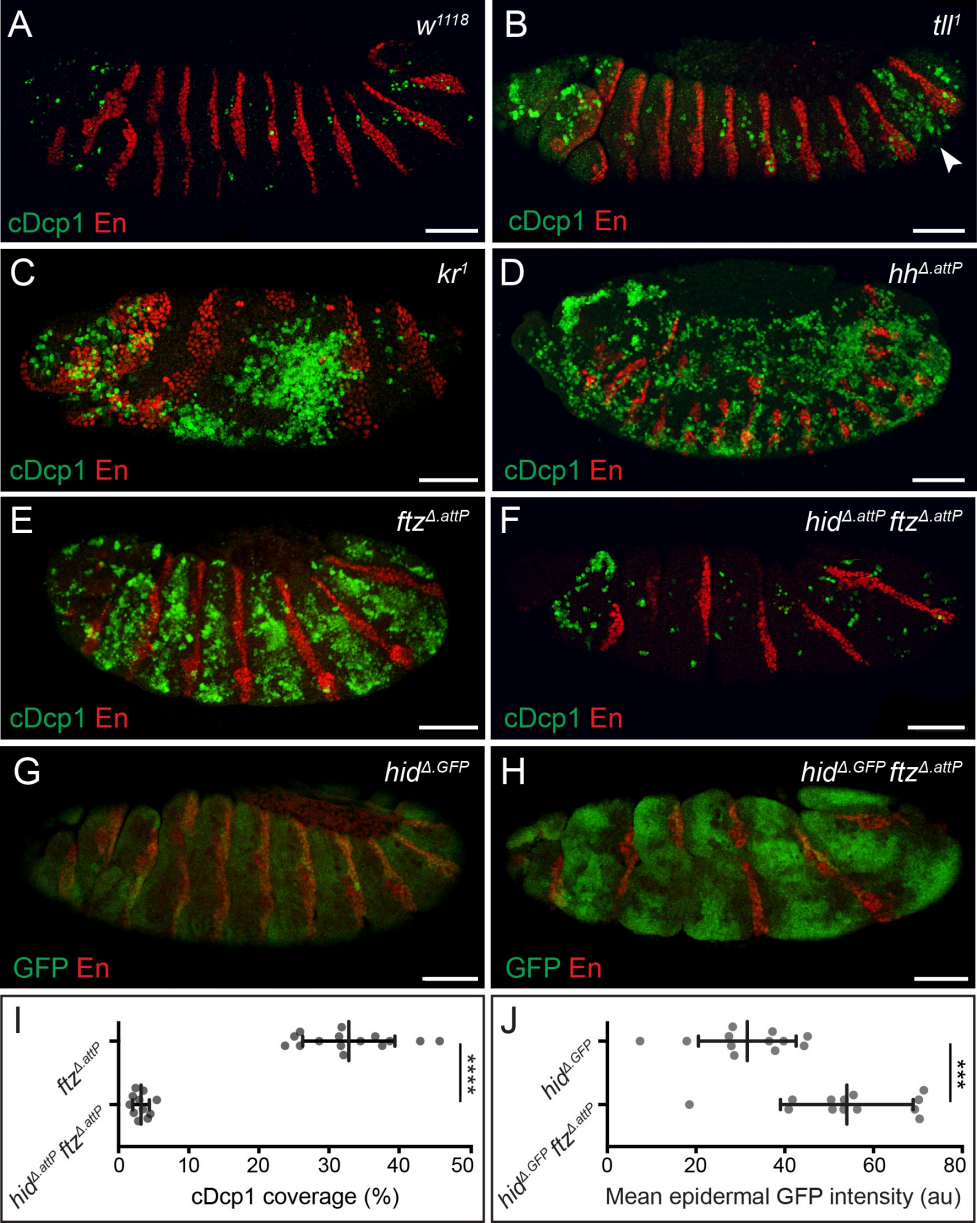
Widespread apoptosis in segmentation mutants is mediated by *hid*. (A–E) cDcp1 immunoreactivity (green), in control (A), *tll* (B), *kr* (C), *hh* (D), and *ftz* (E) stage 13/14 embryos. Segmental enrichment of cDcp1 is detected around the regions where the mutated gene is known to act during normal development. Arrowhead in B indicates the zone of elevated Dcp1 cleavage in the posterior epidermis of *tll*^*1*^ embryos. Anti-Engrailed (En, red) provides a positional reference along the A/P axis throughout. (F) Epidermal cDcp1 immunoreactivity in stage 13 *hid*^*Δ*.*attP*^ *ftz*^*Δ*.*attP*^ double homozygotes is strongly reduced. (G, H) Transcription of *hid*, as assayed with the *hid*^*Δ*.*GFP*^ reporter, is detected at uniform low levels in a wild-type background (G) but is up-regulated in a banded pattern in *hid*^*Δ*.*GFP*^ *ftz*^*Δ*.*attP*^ double homozygotes. Embryos late stage 12. Scale bars 50 μm. (I) Quantification of cDcp1 levels in *ftz*^*Δ*.*attP*^ single mutant and *hid*^*Δ*.*attP*^ *ftz*^*Δ*.*attP*^ double mutant embryos. (J) Quantification of mean epidermal GFP intensity values throughout the epidermis of *hid*^*Δ*.*GFP*^ single mutant and *hid*^*Δ*.*GFP*^ *ftz*^*Δ*.*attP*^ double mutant embryos. In graphs, means are shown, and error bars display standard deviation (****p < 0.0001, ***p < 0.001, unpaired Student *t* test). The underlying data for (I) and (J) can be found in S1 Data. A/P, anterior/posterior; cDcp1, cleaved Death caspase-1; *ftz*, *fushi-tarazu*; GFP, green fluorescent protein; *hh*, *hedgehog*; *hid*, *head involution defective*; *kr*, *krüppel*; *tll*, *tailless*.

Consistent with an earlier report [13], extensive apoptosis was detected in *Drosophila* embryos lacking genes acting at various steps of the segmentation cascade, including mutants of the terminal gene *tailless* (*tll*), the gap gene *krüppel* (*kr*), the pair-rule gene *fushi-tarazu* (*ftz*), or the segment polarity gene *hedgehog* (*hh*) (Fig 1A–1E). In each instance, cleaved Death caspase-1 (Dcp1), a marker of apoptosis, was strongly enriched in the areas where the mutated patterning gene is known to be required during normal development. For example, in a null *ftz* mutant generated for this study (*ftz*^*Δ*.*attP*^), seven bands of apoptotic cells appeared in the segments where Ftz is normally required in the wild type (Fig 1E, see also [12]). Thus, in *ftz*^*Δ*.*attP*^ mutants, alternate segments undergo massive apoptosis, while almost no cell death is detected in the remaining, normally patterned segments, which therefore serve as a useful control. For this reason, *ftz* mutants were selected as a prototypical condition to investigate mispatterning induced apoptosis.

Among the four known proapoptotic genes of *Drosophila*, *hid* is the key mediator of apoptosis in *bicoid* mutant embryos [13]. To assess the involvement of *hid* in *ftz* mutants, we created a clean *hid* allele (*hid*^*Δ*.*attP*^) by replacing the first coding exon, which encodes the IAP-binding motif, with an *attP* integrase site [16–18]. This mutation was recombined with *ftz*^*Δ*.*attP*^, and the resulting double mutant embryos were stained with anti-cleaved Dcp1. Although some signal remained in the head region, immunoreactivity was much lower than in single *ftz*^*Δ*.*attP*^ mutants (Fig 1F and 1I). In contrast, abundant apoptosis was detected in *ftz*^*Δ*.*attP*^ mutants lacking the closely related IAP antagonists *rpr* and *skl* (S1 Fig). We conclude that, as in *bicoid* mutants, *hid* is the main mediator of apoptosis in *ftz* mutant embryos. Thus, in the absence of Hid, cells that would normally be eliminated are forced to contribute to the final pattern. To assess the fate of these “undead cells”, *hid^Δ.attP^ ftz^Δ.attP^* double mutants were allowed to undergo terminal differentiation into first instar larvae, and the pattern of denticles was examined. Alternate denticle belts were missing while numerous nondescript ectopic denticles appeared at the posterior of each remaining belt (S2 Fig). In addition, the cuticle appeared particularly convoluted, suggesting that tissue size is at least partially restored compared to the single mutant. Therefore, as previously suggested for *bicoid* mutants [13], it appears that undead cells can contribute to the epidermis but are unable to adopt their normal fate.

To visualize the expression of *hid* in mispatterned embryos, we generated an authentic transcriptional reporter by integrating a cDNA encoding green fluorescent protein (GFP) into the *attP* site of *hid*^*Δ*.*attP*^ [16]. These animals will be referred to as *hid*^*Δ*.*GFP*^ to indicate that this genetic modification creates a null allele (Δ) as well as a fluorescent reporter (GFP). To increase signal intensity, the *hid*^*Δ*.*GFP*^ sensor was examined in homozygous conditions throughout, which has the dual benefit of doubling the copy number of the GFP reporter in the genome whilst simultaneously preventing apoptosis, which could eliminate cells before the GFP signal has time to accumulate. In homozygous *hid*^*Δ*.*GFP*^ embryos, a weak GFP signal could be detected throughout the epidermis, suggesting that low-level *hid* expression occurs at subapoptotic levels during normal development (Fig 1G). In *hid*^*Δ*.*GFP*^ *ftz*^*Δ*.*attP*^ double homozygotes, increased levels of GFP fluorescence were detected in broad stripes, resembling the bands of caspase immunoreactivity seen in *ftz*^*Δ*.*attP*^ single mutants (Fig 1H and 1J). Time-lapse imaging showed that striped GFP fluorescence arises around stage 11 of embryogenesis (S1 Movie), approximately 4 hours after the time when pair-rule genes contribute to segmental patterning [19,20]. Likewise, cleaved Dcp1 immunoreactivity became only detectable at stage 11 in homozygous *ftz*^*Δ*.*attP*^ embryos (S3A–S3C Fig). Therefore, loss of *ftz* appears to trigger *hid* expression and apoptosis with a delay, well after Ftz has fulfilled its patterning role in wild-type embryos. The same delay was noted in *bicoid* mutants [13], suggesting that it could be a general feature of segmentation mutants.

### EGFR signaling and *hid* expression are anticorrelated in the embryonic epidermis

**Fig 2:**
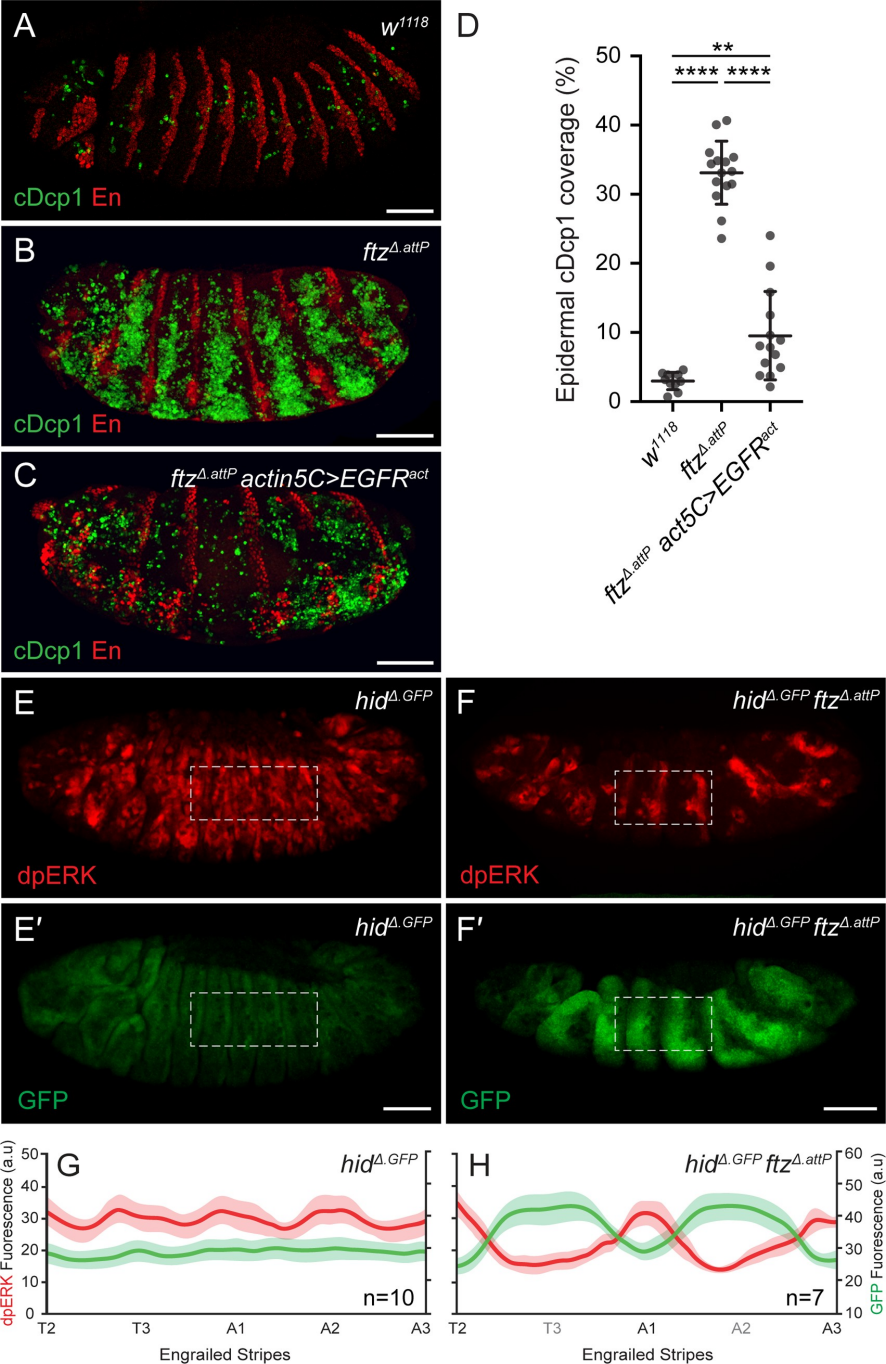
Loss of EGFR signaling and *hid* up-regulation in *ftz* mutants. (A–D) cDcp1 immunoreactivity in control *w*^*1118*^ (A), *ftz*^*Δ*.*attP*^ (B), and *ftz*^*Δ*.*attP*^ *actin5C-*Gal4, UAS–*EGFR*^*act*^ (C) stage 13/14 embryos. (D) Quantification of mean cDcp1 levels for genotypes shown in A–C. Error bars display standard deviation (***p < 0.001, **p < 0.01, unpaired Student *t* test). (E, F) dpERK/GFP immunoreactivity in stage 13 *hid*^*Δ*.*GFP*^ single mutant (E, E′) and *hid*^*Δ*.*GFP*^ *ftz*^*Δ*.*attP*^ double mutant embryos (F, F′). Dashed line indicates representative region of interest for quantification in panels G and H. Scale bars 50 μm. (G, H) Mean dpERK (red) and GFP (green) fluorescence intensity profiles in control *hid*^*Δ*.*GFP*^ (G) and *hid*^*Δ*.*GFP*^ *ftz*^*Δ*.*attP*^ (H) samples. Engrailed expression stripes were used as a spatial marker along the A/P axis (see material and methods). Shaded areas depict standard error of the mean. The underlying data for (D), (G) and (H) can be found in S1 Data. cDcp1, cleaved Death caspase-1; dpERK, phosphorylated Extracellular signal–regulated kinase; EGFR, epidermal growth factor receptor; *ftz*, *fushi-tarazu*; GFP, green fluorescent protein; *hid*, *head involution defective*; UAS, upstream activation sequence; *w*^*1118*^, *white*^*1118*^.

Mismatched or nonsense cell fates could conceivably be recognized through local cell interactions. With the aim of identifying the relevant mediator, we used the ubiquitous *actin5C*-Gal4 driver to overexpress modulators of major signaling pathways in a *ftz*^*Δ*.*attP*^ mutant background and stained the resulting embryos with anti-cleaved Dcp1 (summarized in S1 Table). One manipulation, overexpression of an activated form of the *Drosophila* epidermal growth factor receptor (*EGFR*^*act*^, [21]), significantly reduced cleaved Dcp1 immunoreactivity (Fig 2A–2D). Since EGFR signaling is required for survival of the cells that secrete naked cuticle [22] and since EGFR signaling has been previously implicated in suppressing *hid* activity [23,24], we set out to determine whether the excess apoptosis and up-regulation of *hid* in *ftz* mutants could be accounted for by a loss of EGFR activity.

If EGFR signaling is needed to keep *hid* expression off, we would expect *hid* expression to be up-regulated in EGFR mutants. Indeed, strong ubiquitous GFP fluorescence was observed in embryos homozygous for both *EGFR*^*F3*^, an amorphic allele, and *hid*^*Δ*.*GFP*^ (S4A and S4B Fig). This suggests that most, if not all, epidermal cells require EGFR signaling to keep *hid* expression low. Conversely, the prosurvival activity of EGFR signaling appears to be largely mediated by repression of *hid* activity since almost no apoptosis was detected in *EGFR*^*F3*^ *hid*^*Δ*.*attP*^ double mutants (S4D Fig). In *EGFR*^*F3*^ single mutant embryos, cell death was widespread (S4C Fig) but did not occur until embryonic stage 11 (S3D–S3F Fig), the same time at which apoptosis becomes detectable in *ftz* mutants. We conclude that, in the absence of EGFR signaling, most cells of the epidermis up-regulate *hid* expression and undergo apoptosis around mid-embryogenesis.

To visualize EGFR signaling activity in mispatterned *ftz* embryos, we stained embryos with an antibody that recognizes phosphorylated extracellular signal–regulated kinase (dpERK) [25]. As validation, we first stained *EGFR*^*F3*^ homozygotes. No immunoreactivity was detectable above background levels (S5 Fig), a strong indication that EGFR is the principal contributor of ERK phosphorylation in the embryonic epidermis. Anti-dpERK was next used to compare the patterns of EGFR signaling activity in *ftz*^*Δ*.*attP*^ mutant and control embryos. The *hid*^*Δ*.*GFP*^ allele (homozygous) was included to allow simultaneous assessment of *hid* expression and to avoid possible confounding effects of apoptosis on signaling activity. In control *hid*^*Δ*.*GFP*^ embryos, dpERK immunoreactivity was observed in a wide range of tissues, including the epidermis but also the peripheral nervous system, oenocytes, and the tracheal precursors (Fig 2E) [26–29]. In the epidermis, dpERK immunoreactivity was detectable in nearly all cells, though not uniformly so. Signal intensity followed a broadly undulating pattern along the anterior/posterior (A/P) axis, with smooth peaks near segment boundaries and intervening shallow troughs (Fig 2G). Little GFP fluorescence was detectable in the epidermis (Fig 2E′ and 2G), confirming that *hid* transcription (as reported by *hid*^*Δ*.*GFP*^) remains low in normally patterned embryos (see also Figs 1G and S3A). In the absence of *ftz* activity, dpERK immunoreactivity dropped significantly in seven broad bands (Fig 2F and 2H), leaving only half the number of signaling peaks intact. At the same time, a complementary pattern of GFP fluorescence (from the *hid*^*Δ*.*GFP*^ reporter) appeared (Fig 2F and 2H). We conclude that, in *ftz* mutant embryos, the transcriptional activation of *hid* increases in regions where EGFR signaling activity fails to reach the threshold level required for epidermal cell survival.

**Fig 3:**
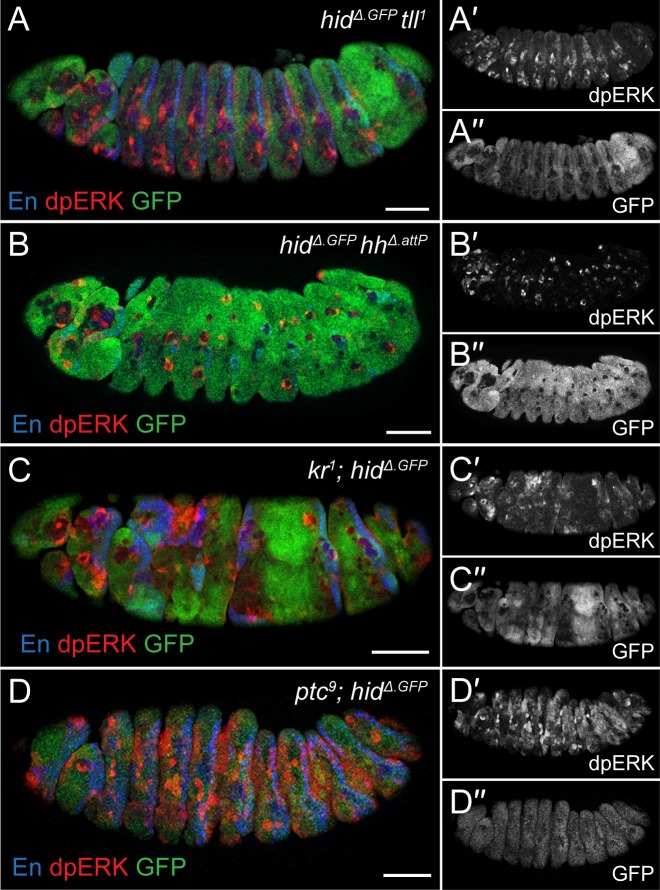
*hid* and dpERK are anticorrelated in various patterning mutants. (A–D) GFP and dpERK immunoreactivity in *hid*^*Δ*.*GFP*^ *tll*^*1*^ (A), *hid*^*Δ*.*GFP*^ *hh*^*Δ*.*attP*^ (B), *kr*^*1*^*; hid*^*Δ*.*GFP*^ (C), and *ptc*^*9*^*; hid*^*Δ*.*GFP*^ (D) stage 13 double mutants. In each instance, dpERK intensity and *hid*^*Δ*.*GFP*^ expression are anticorrelated. Scale bars 50 μm. dpERK, phosphorylated extracellular signal–regulated kinase; GFP, green fluorescent protein; *hh*, *hedgehog*; *hid*, *head involution defective*; *kr*, *krüppel*; *ptc*, *patched*; *tll*, *tailless*.

Other segmentation mutants, including *tll*, *kr*, and *hh*, also displayed anticorrelated *hid*^*Δ*.*GFP*^ reporter activity and dpERK immunoreactivity (Fig 3A–3C). Importantly, the pattern of *hid* expression in these mutants corresponded to that of apoptosis (as shown in Fig 1A–1D). Embryos lacking another segmentation gene, *patched*, were particularly informative, as they had increased dpERK immunoreactivity and did not up-regulate *hid*^*Δ*.*GFP*^ (Fig 3D). Crucially, they also showed no excess apoptosis, as assayed with anti-cleaved Dcp1 (S6 Fig). This observation confirms the tight correlation between EGFR signaling and the absence of *hid* expression. It also shows that not all instances of patterning error are associated with apoptotic cell death.

### Segmentally expressed EGF ligands coordinate apoptosis with the patterning cascade

EGFR signaling has previously been widely implicated in cell survival in the embryo and other tissues [22,30,31]. Known *Drosophila* EGFR ligands include the TGF-α homologues Spitz, Keren, and Gurken, which require activation by Rhomboid proteases [32–35], as well as the Rhomboid-independent neuregulin-like ligand Vein [36]. To assess the nature of the ligands involved in the survival of embryonic epidermal cells, we first examined embryos homozygous for a deficiency (*Df* ^*rho*-1,3^) spanning the *rhomboid* (*rho*-1) and *roughoid* (*rho*-3) loci. In this background, a relatively mild apoptotic phenotype was observed (S7B Fig). Likewise, homozygous *vein* (vn) mutants were also characterized by a relatively minor increase in the number of apoptotic cells compared to wild-type embryos (S7C Fig). In contrast, simultaneous removal of both ligand systems, in *Df* ^*rho*-1,3^ *vn*^*L6*^ double homozygotes, led to widespread apoptosis (S7D Fig), consistent with the severe cuticle phenotype previously reported [22]. We conclude that multiple ligands act redundantly to elicit the EGFR signaling landscape that, at mid-embryogenesis, prevents apoptosis.

**Fig 4:**
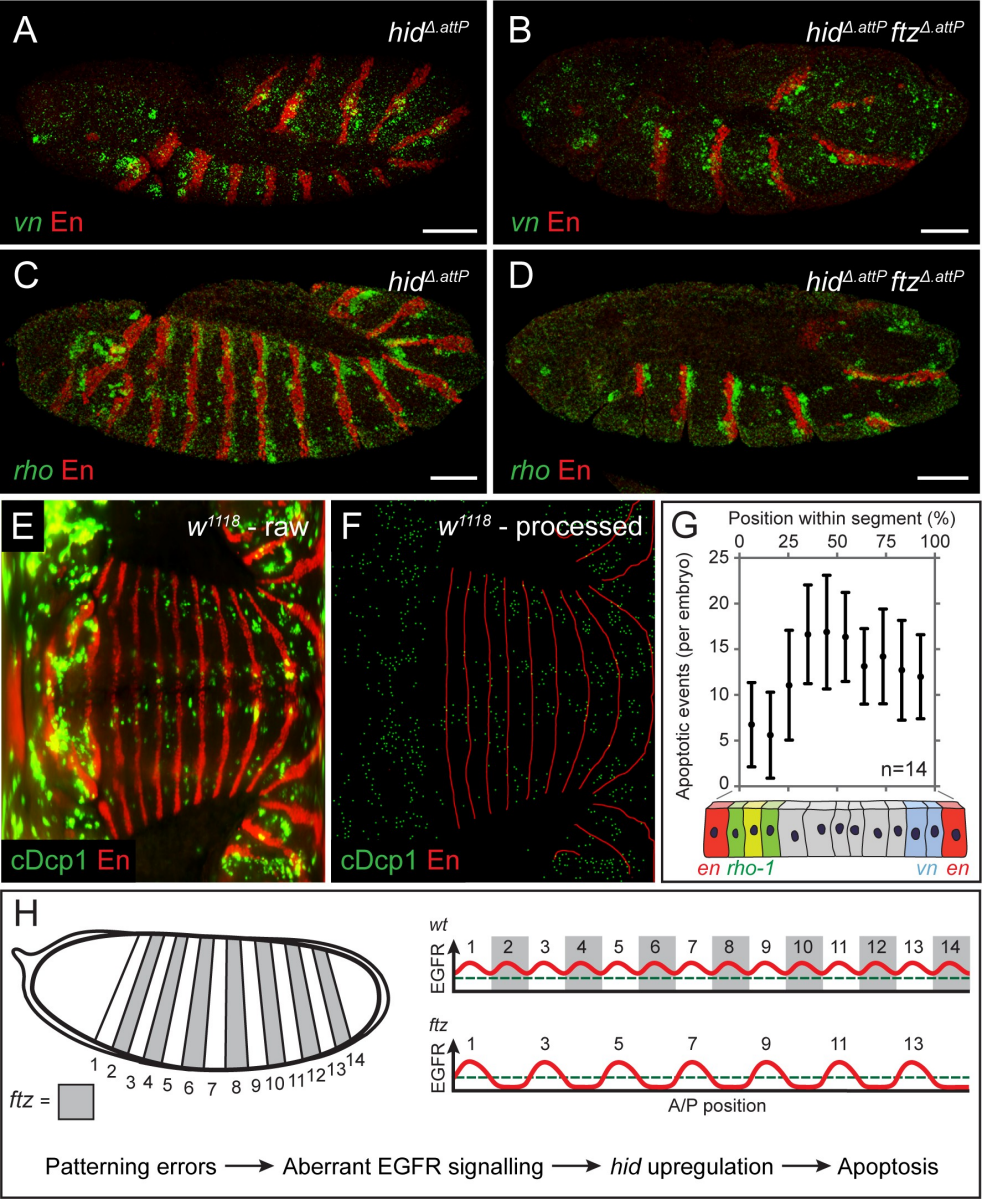
Patterned expression of *vn* and *rho*-1 ensure epidermal cell survival. (A, B) Expression of *vn* (green) in control *hid*^*Δ*.*attP*^ (A) and *hid*^*Δ*.*attP*^ *ftz*^*Δ*.*attP*^ (B) stage 12 homozygotes, as detected by fluorescent in situ hybridisation. *hid*^*Δ*.*attP*^ is included in the background to avoid any confounding effects of apoptosis. (C, D) Expression of *rho*-1 (green) in *hid*^*Δ*.*attP*^ (C) and *hid*^*Δ*.*attP*^ *ftz*^*Δ*.*attP*^ (D) stage 12 homozygotes, as detected by fluorescent in situ hybridisation. Scale bars 50 μm. (E) Representative raw mSPIM image of a stage 12 *w*^*1118*^ embryo (control) stained with anti-cDcp1 and anti-En (red). Anterior is to the left and posterior to the right, with the ventral midline running horizontally through the middle. (F) Processed version of the image shown in E. cDcp1-positive cells were segmented and reduced to individual pixels (green). Similarly, stripes of *en* expression were reduced to a skeleton (red) to allow the position of each apoptotic cell to be mapped along the A/P axis of the segment and thus to derive a relative position applicable to all segments. (G) Histogram displaying the frequency of apoptosis along the A/P axis of the segment. Illustration of the *en*, *rho*-1, and *vn* expression domains is included to indicate the approximate sites of ligand production. Error bars show standard deviation. (H) Model explaining the pattern of apoptosis in *ftz* and other segmentation mutants. In normal development, the segmental expression of EGFR ligands maintains signaling activity above the threshold level required for cell survival (green dashed line). When patterning errors disrupt the landscape of EGFR activity, signaling falls below the survival threshold, triggering *hid* up-regulation and patterned apoptosis. The underlying data for (G) can be found in S1 Data. A/P, anterior/posterior; cDcp1, cleaved Death caspase-1; EGFR, epidermal growth factor receptor; *en*, *engrailed*; *ftz*, *fushi-tarazu*; *hid*, *head involution defective*; mSPIM, multiview single plane illumination microscopy; *rho*-1, *rhomboid*; *vn*, *vein*; *w*^*1118*^, *white*^*1118*^.

In agreement with previously described expression patterns [36,37], *vn* and *rho*-1 were found to be expressed in a generally segmental manner in wild-type embryos (Fig 4A and 4C). Thus, peaks of ligand production correspond roughly to the areas of high-dpERK immunoreactivity seen around segmental borders (Fig 2G). We therefore considered whether changes in ligand production could account for the observed changes in EGFR signaling in patterning mutants. The pattern of *rho*-1 expression is expected to depend on the segmentation network since it is determined by segment polarity genes [37,38], which are themselves regulated by upstream pair-rule and gap genes. It is likely that the segmentation network similarly controls *vn* expression. Therefore, as expected, peaks of *vn* and *rho*-1 expression failed to appear in alternate segments of *ftz*^*Δ*.*attP*^ mutants (Fig 4B and 4D), foreshadowing the loss of dpERK immunoreactivity (Fig 2F), *hid* up-regulation (Fig 1H), and apoptosis (Fig 1E). Importantly, the same logic can explain the pattern of apoptosis in other segmentation mutants, which are all expected to affect the pattern of EGFR ligand production within their realm of action. It also neatly explains the absence of apoptosis in *patched* (*ptc*) mutants, in which ectopic peaks of *rho-1* expression arise [37].

During normal development, EGFR signaling is lowest in cells located around the middle of each segment, which are likely to be exposed to the lowest amounts of ligand (Fig 2G). We asked whether these cells are particularly susceptible to apoptosis. To map the pattern of apoptosis along the A/P axis, we performed in toto multiview single plane illumination microscopy (mSPIM) on wild-type embryos stained with anti-cleaved Dcp1 and anti-Engrailed, which marks the anterior edge of each segment boundary. This approach allowed us to visualize, at a given time point, all epidermal apoptotic events, which are relatively rare during normal development (see Fig 1A and Fig 2A). Raw image files were generated (Fig 4E), segmented (Fig 4F), and the position of each caspase-positive cell was then mapped and normalized relative to the nearest anterior and posterior segment boundary. These data were then tabulated in a histogram displaying the frequency of apoptosis at various positions along the A/P axis within each segment (Fig 4G). Cell death was relatively infrequent near segment boundaries, where *vn* and *rho*-1 are most highly expressed (Fig 4A–4D), while apoptosis was more abundant in the middle of the intervening areas. We conclude that, in the cells that are most distant from a source of ligand, EGFR signaling activity falls near, and occasionally below, the threshold level needed to protect against apoptosis. Cells in these regions are therefore more likely to commit to an apoptotic cell fate.

Despite the observed correlation between the distance to segment boundaries and the rate of apoptosis in wild-type embryos, it is clear that cells located near the sources of EGFR ligand production can still undergo apoptosis. In this context, it is worth pointing out that only a subset of embryonic apoptotic events are regulated by the EGFR–Hid axis, since residual apoptosis is still detected in *hid* mutants [39], and Hid-independent apoptotic processes have been characterized in the *Drosophila* embryo [40]. Therefore, it is likely that additional factors, besides EGFR signaling, contribute to the control of apoptosis in the embryonic epidermis.

## Discussion

Excess apoptosis in patterning mutants gives the impression that mispatterned cells are recognized as deleterious and eliminated during development. We have shown that excess apoptosis in embryonic patterning mutants can be explained by the requirement of EGFR signaling for cell survival and the fact that the segmentation cascade culminates in the segmental production of EGF ligands (model summarized in Fig 4H). Thus, in segmentation mutants, sources of EGF ligand production are lost, leaving remaining sources unable to reach all intervening cells. This results in tissue loss until all cells are brought back within signaling range, restoring a “normal” distance between segment boundaries. EGFR signaling has previously been linked to compartment size regulation in the embryo [41]. Specifically, it was proposed that epidermal cells in the posterior regions of each segment compete for a finite amount of Spitz emanating from an anterior source. Our results are consistent with an extended version of this model, whereby multiple EGFR ligands (including Spitz and Vein) produced primarily at either side of the segment boundary control the size of entire segments, not just posterior compartments. The pattern of apoptosis in wild-type embryos (Fig 4G) suggests that segments are slightly oversized upon completion of patterning and that the extra cells are eliminated as a result of subthreshold EGFR signaling and *hid* activation. Accordingly, there is no need to invoke the existence of a mechanism that recognizes and eliminates mis-specified cells, and mispatterning-induced cell death could be the byproduct of a size control system.

As we have shown, the lack of EGFR signaling triggers *hid* expression only at stage 11, when proliferation is largely complete [42]. This would serve as a particularly suitable time for a size control checkpoint and pruning of excess cells would be an effective means of ensuring reproducible segment dimensions in a fast-growing embryo with no compensatory proliferation. Similar prosurvival signaling by limited ligand diffusion from specific locations could form the basis of tissue size sensing and explain mispatterning-induced apoptosis in a variety of developmental contexts. It is intriguing that, while EGFR signaling over a relatively low threshold is required for cell survival, higher signaling activity (as occurs near the segment boundary) contributes to the specification of pattern elements (i.e., denticles) [22,37,43–45]. Thus, the same ligands contribute to both patterning and cell survival, perhaps ensuring a tight coordination of these two essential developmental activities.

## Materials and methods

### *Drosophila* stocks and husbandry

Flies were maintained at 25°C on standard fly food containing yeast, agar, and cornmeal. *w*^*1118*^ was used as a wild-type control throughout. Fly stocks used in this study are listed in Table 1.

**Table 1:** *Drosophila* stocks used in the present study.

Genotype	Source	Identifier
*actin5C*-GAL4/TM6B	BDSC	FBst0003954
*Df*^*(3L)BSC178*^/TM6B (*Df* ^*rho*-1, 3^)	BDSC	FBst0009609
*Df*^*(3L)XR38*^/TM3	Irene Miguel-Aliaga	N/A
*EGFR*^*F3*^/CyO	BDSC	FBst0034043
*ftz*^Δ.*attP*^/TM3	This study	N/A
*hh*^Δ.*attp*^/TM3	Cyrille Alexandre [18]	N/A
*hid*^Δ.*attp*^/TM3	This study	N/A
*hid*^Δ.*GFP*^/TM3	This study	N/A
*kr*^*1*^/CyO	BDSC	FBst0003494
*ptc*^9^/CyO	BDSC	FBst0003377
*tll*^1^/TM3, Sb	BDSC	FBst0002729
UAS-*tkv*^*act*^	Markus Affolter	N/A
UAS-*hh*	Peter Lawrence	N/A
UAS-*EGFR*^*act*^	BDSC	FBst0009534
UAS-*p53*^*DN*^	BDSC	FBti0040583
UAS-*puc*	Enrique Martin-Blanco	N/A
UAS-*upd*	David Strutt [46]	FBtp0011049
UAS-*wg*	Xavier Franch-Marro	N/A
UAS-*yki*.GFP	Nic Tapon	N/A
*vn*^L6^/TM3	Amanda Simcox	N/A
*w*^*1118*^	BDSC	FBst0003605

**Abbreviations:** BDSC, Bloomington *Drosophila* Stock Centre; CyO, Curly of Oster; *Df*, *deficiency*; EGFR, epidermal growth factor receptor; *ftz*, *fushi-tarazu*; GFP, green fluorescent protein; *hh*, *hedgehog*; *kr*, *krüppel*; *ptc*, *patched*; *puc*, *puckered*; *rho*-1, *rhomboid*-1; *rho*-3, *roughoid*; Sb, *stubble*; *tkv*, *thickveins*; *tll*, *tailless*; UAS, upstream activation sequence; *upd*, *unpaired*; *vn*, *vein*; *w*^*1118*^, *white*^*1118*^; *wg*, *wingless*; *yki*, *yorkie*.

To identify regulators of apoptosis in mispatterned cells, a collection of UAS lines was assembled. Each expressed a gene that modulates the activity of a signaling pathway in the presence of Gal4 (S1 Table). These so-called UAS lines were crossed to the null *ftz* allele *ftz*^*Δ*.*attP*^, and male progeny containing both the *ftz* allele and the UAS transgene were crossed to females bearing the ubiquitous *actin5C*-Gal4 driver recombined to *ftz*^*Δ*.*attP*^. The embryos produced from this cross were then stained for cleaved Dcp1.

### Generation of transgenic flies

Accelerated “ends out” homologous recombination was used to generate the *hid*^*Δ*.*attP*^ and *ftz*^*Δ*.*attP*^ alleles (as described in [18]). Homology arms of approximately 2 kb were PCR amplified from genomic DNA. These fragments were cloned into a targeting vector (pTV^Cherry^) containing a GMR > *white* eye marker and an *attP* landing site flanked with FRT sites. UAS-*rpr* was included downstream of the 3′ homology arm. Targeting vectors were integrated into the genome via P element–mediated transformation to generate so-called donor lines. To release the homology cassette from its genomic location, donor flies were crossed to *hs*-FLP and heat shocked at 24-hour intervals throughout larval development. Adult female progeny with mottled eye color (indicating the presence of both the targeting vector and *hs*-FLP constructs) were crossed to the strong *ubi*-Gal4 driver to eliminate individuals that had either failed to excise the homology cassette or had undergone an unsuccessful recombination event. Resultant *white*^*+*^ candidates were then isolated and verified with PCR.

To generate the *hid*^*Δ*.*GFP*^ reporter, a GFP cDNA was cloned into the RIV^Cherry^ vector [18], which contains an attB sequence and a *3xPax3-cherry* selection marker. This construct was injected into *hid*^*Δ*.*attP*^ embryos along with a source of ΦC31 integrase, and progeny were screened for Cherry expression. Rainbow Transgenic Flies, Inc. (Camarillo, California, United States) and Bestgene, Inc. (Chino Hills, California, United States) were used for embryo injection services.

### Embryo collection

Embryos were collected overnight, transferred to baskets, and washed with water. Embryos were then dechorionated using 75% bleach for 3 minutes and washed again. After drying on a paper towel, embryos were transferred with a brush to microcentrifuge tubes containing equal volumes of heptane and 6% formaldehyde in phosphate buffered saline (PBS). Fixation was performed for 25 minutes on a rotating platform. The fixative was then removed and replaced with 1 ml methanol. Embryos were shaken vigorously for 45 seconds to remove the vitelline membrane, and the heptane was removed. Embryos were washed twice more in methanol and moved to −20°C for long-term storage.

### Immunofluorescence

Fixed embryos were rehydrated in 0.3% triton in PBS (PBT) and blocked for 30 minutes at room temperature in 4% bovine serum albumin in PBT. Embryos were incubated with primary antibody at 4°C overnight, washed for 1 hour in PBT at room temperature, and then incubated in a solution of Alexa Fluor 488, 568, or 633 conjugated species appropriate secondary antibodies (Thermo Fisher Scientific, 1:1,000) for 2 hours. Further washes (1 hour) were conducted before samples were mounted in Vectashield containing DAPI (Vector Laboratories). Primary antibodies used in this study are listed in Table 2.

**Table 2:** Primary antibodies used in the present study.

Name	Source	Identifier
mouse monoclonal anti-Engrailed	DSHB	AB_528224
chicken polyclonal anti-GFP	Abcam	AB_300798
rabbit polyclonal anti-cleaved Dcp1	Cell Signalling Technologies	AB_2721060
rabbit polyclonal anti-dpERK	Cell Signalling Technologies	AB_331772
rat monoclonal anti-Cadherin	DSHB	AB_528120

**Abbreviations:** Dcp1, Death caspase-1; dpERK, phosphorylated extracellular signal–regulated kinase; DSHB, Developmental Studies Hybridoma Bank; GFP, green fluorescent protein.

### Confocal microscopy

Confocal microscopy was conducted with a Leica SP5 confocal microscope using a 20x (Leica, NA 0.7) or 40x (Leica, NA 1.25) oil immersion objective. Typically, 8 confocal planes were imaged at 1.8 μm intervals and processed using Fiji (NIH) and Photoshop (Adobe).

### Live confocal imaging

Samples were prepared using the hanging drop method described in [47]. A small drop of oxygen permeable Voltalef 10S oil was spotted onto a cover slip. A dechorionated embryo was then placed inside the oil and oriented with a 27-G hypodermic needle. The sample was then inverted so that the oil containing the sample hung below the cover slip. Images were acquired from above at 10 minute intervals using a Leica SP5 confocal microscope and processed using imageJ (NIH) and Photoshop (Adobe) as described above.

### mSPIM imaging

Fluorescently labeled samples were imaged on a multiview light sheet microscope [48]. The optics consisted of two detection and illumination arms. Here, each detection arm formed an epifluorescence microscope using in sequence: a water-dipping lens (Apo LWD 25x, NA 1.1, Nikon Instruments, Inc.), a filter wheel (HS-1032, Finger Lakes Instrumentation LLC) equipped with emission filters (BLP01-488R-25, BLP02-561R-25, Semrock, Inc.), a tube lens (200 mm, Nikon Instruments, Inc.), and sCMOS camera (Hamamatsu Flash 4.0 v2.0). The imaging produced an effective pixel size of 0.26 μm. The illumination arms each consisted in sequence: a water-dipping objective (CPI Plan Fluor 10x, NA 0.3, Nikon Instruments, Inc.), a tube lens (200 mm, Nikon Instruments, Inc.) a scan lens (S4LFT0061/065, Sill optics GmbH & Co. KG), and a galvanometric scanner (6215h, Cambridge Technology, Inc.). The illumination arms were fed by a combination of lasers (06-MLD 488 nm, Cobolt AB, and 561 LS OBIS 561 nm, Coherent, Inc.). Samples were translated through the resulting light sheet using a linear piezo stage (P-629.1CD together with E-753 controller). Multiple rotation views were achieved using a rotational piezo stage (U-628.03 with C-867 controller) in combination with a linear actuator (M-231.17 with C-863 controller, all Physik Instrumente GmbH and Co. KG). Microscope operation was done using Micro Manager [49], running on a Super Micro 7047GR-TF Server, with 12 Core Intel Xeon 2.5 GHz, 64-GB PC3 RAM, and hardware Raid 0 with 7 2.0 TB SATA drives. Four views with a rotational offset of 90° were recorded at 1 μm sectioning. Fusion of individual views is based on a diagnostic specimen used to determine an initial guess for an affine transformation that maps each view into a common frame of reference. Together with the data, this initial guess was used with a rigid image registration algorithm [50] to fuse individual views and resulted in isotropic resolution of 0.26 μm in the final registered image. ImSAnE [51] was used to obtain tissue cartographic projections showing global maps of the embryo surface of interest.

### Fluorescent in situ hybridisation

Digoxigenin labeled antisense RNA probes were synthesized from Gold Collection (Berkeley Drosophila Genome Project) clones containing full length versions of the *vn* or *rho*-1 cDNAs (clone number LP21849 and LD06131, respectively). Vectors were linearized with the EcoRI restriction enzyme and used as a template for in vitro transcription as per manufacturer instructions (Roche, SP6/T7 DIG RNA-labeling kit). Fixed embryos were bathed in 3% hydrogen peroxide in methanol for 15 minutes to remove endogenous peroxidase activity and rinsed extensively in PBT. Embryos were then incubated for 3 minutes in 10 μg/ml proteinase K in PBT and washed in 2 mg/ml Glycine in PBT. Samples were next washed in a hybridization buffer before the relevant probe was added, and the samples incubated overnight at 55°C. The next day, the embryos were washed once more in PBT and incubated overnight at 4°C with a sheep anti-DIG antibody diluted at 1:2,000. On the third day, embryos were washed and incubated with a biotinylated anti-sheep secondary antibody, and the final signal was developed using a Tyramide-FITC signal amplification kit (Thermo Fisher Scientific). Standard immunostaining was then performed on these samples, using the protocol outlined above.

### Quantification and statistical analysis

To measure fluorescence intensity along the A/P axis, rectangular regions of interest with a fixed height of 75 μm were drawn over four contiguous segments. In each instance, efforts were made to ensure the same embryonic regions were analyzed between samples. GFP (from *hid*^Δ.GFP^), dpERK, and Engrailed intensity profiles were generated across the regions of interest in Fiji [52], and these data were imported into MATLAB (Mathworks) for further analysis. GFP and dpERK intensity profiles were fitted to the peaks of *engrailed* expression (i.e., segment boundaries) along the A/P axis to normalize data to segment width, which can vary between samples. This was achieved by creating 50 evenly spaced intervals along the A/P axis of each segment before extracting the raw data values and averaging the resulting numbers across all samples analyzed. standard error of the mean was calculated for each point, and data was plotted using the boundedline.m function developed by Kelly Kearney and downloaded from the MATLAB stack exchange.

To measure the frequency of apoptosis along the segmental A/P axis, the Engrailed, and cleaved Dcp1 channels were separated from the raw mSPIM image files and then segmented using the open source iLastik software (University of Heidelberg). Segmented images were imported into ImageJ and reduced to single pixel lines and points using the software’s inbuilt skeletonization algorithms. From these skeletons, individual segment boundaries were manually classified and masks were generated that covered each of the segments to be analyzed. Processed files were then transferred to MATLAB for further analysis. Distances were calculated from each Dcp-1 positive pixel to the nearest anterior and posterior Engrailed positive pixel using a k-nearest neighbors algorithm, and these measurements were used to determine the position of each apoptotic cell along the segmental A/P axis as a percentage of segment width. Cells located in the immediate vicinity of the ventral midline (defined as 20% of linear Dorsal to Ventral distance) were excluded from the analysis to avoid apoptotic figures in the developing nervous system. The retained data were collated and tabulated as a histogram.

## Supporting information

S1 Fig Apoptosis in *ftz* mutants does not require *rpr*. (A) Schematic representation of the *Drosophila* H99 locus showing the genes encoding the four major IAP antagonists. The *Df*^*(3L)XR38*^ deficiency, which removes the *rpr* and *skl* genes, is highlighted in blue. (B, C) Cleaved Dcp1 immunoreactivity in stage 12 *ftz*^*Δ*.*attP*^ (B) and *ftz*^*Δ*.*attP*^ *Df*^*(3L)XR38*^ homozygotes (C). Scale bars 50 μm. *Df*, *deficiency*; *ftz*, *fushi-tarazu*; IAP, inhibitor of apoptosis protein; *rpr*, *reaper*; *skl*, *sickle*.
(TIF)

S2 Fig “Undead cells” undergo terminal differentiation in *ftz* mutants that cannot undergo apoptosis. (A, B) Cuticle preparations of *ftz*^*Δ*.*attP*^ (A) and *hid*^*Δ*.*attP*^ *ftz*^*Δ*.*attP*^ (B) embryos. In *ftz*^*Δ*.*attP*^ homozygotes, remaining denticle belts have near wild-type morphology. In *hid*^*Δ*.*attP*^ *ftz*^*Δ*.*attP*^ homozygotes, the remaining denticle belts are expanded posteriorly with multiple rows of nondescript ectopic denticles (B′). *ftz*, *fushi-tarazu*; *hid*, *head involution defective*.
(TIF)

S3 Fig Apoptosis in *ftz* and *EGFR* mutants is first detectable at embryonic stage 11. (A–C) Cleaved Dcp1 immunoreactivity in *ftz*^*Δ*.*attP*^ mutants at embryonic stages 9 (A), 11 (B), and 13+ (C). (D–F) Cleaved Dcp1 immunoreactivity in *EGFR*^*F3*^ mutants at embryonic stages 9 (D), 11 (E), and 13+ (F). In both genotypes, cleaved Dcp1 is first detected in stage 11 and persists throughout the remainder of embryonic development. Scale bars 50 μm. Dcp1, Death caspase-1; EGFR, epidermal growth factor receptor; *ftz*, *fushi-tarazu*.
(TIF)

S4 Fig EGFR signaling maintains epidermal cell survival through repression of *hid*. (A, B) Transcription of *hid*, as assayed with the *hid*^*Δ*.*GFP*^ reporter, in control (A) and *EGFR*^*F3*^ mutant (B) stage 12 embryos. *hid* is up-regulated in most epidermal cells upon loss of EGFR signaling. (C, D) Cleaved Dcp1 immunoreactivity is strongly up-regulated throughout the epidermis in stage 12 *EGFR*^*F3*^ single mutants (C) and this up-regulation is lost in stage 12 *EGFR*^*F3*^; *hid*^*Δ*.*attP*^ double homozygotes (D). Scale bars 50 μm. Dcp1, Death caspase-1; EGFR, epidermal growth factor receptor; GFP, green fluorescent protein; *hid*, *head involution defective*.
(TIF)

S5 Fig dpERK immunoreactivity is markedly reduced upon loss of EGFR signaling. (A, B) dpERK immunoreactivity in control (*EGFR*^+/+^) *hid*^*Δ*.*attP*^ (A) and *EGFR*^*F3*^; *hid*^*Δ*.*attP*^ double homozygotes. Extensive dpERK immunoreactivity is detected in wild-type control embryos (A) and this signal is largely lost in *EGFR*^*F3*^; *hid*^*Δ*.*attP*^ double mutants (B). We take this as evidence that EGFR signaling is the major source of ERK phosphorylation in the embryonic epidermis. Scale bars 50 μm. dpERK, phosphorylated extracellular signal–regulated kinase; EGFR, epidermal growth factor receptor; *hid*, *head involution defective*.
(TIF)

S6 Fig Absence of ectopic apoptosis in *ptc* mutants. (A, B) Cleaved Dcp1 immunoreactivity in control *w*^*1118*^ (A) and *ptc*^*9*^ mutant embryos (B) at embryonic stage 13. No increase in Dcp1 cleavage was detected in *ptc* mutants, despite disruption to the segmental pattern. Scale bars 50 μm. Dcp1, Death caspase-1; *ptc*, *patched*; *w*^*1118*^, *white*^*1118*^.
(TIF)

S7 Fig Vein and Rhomboid act redundantly to maintain epidermal cell survival. (A–D) Cleaved Dcp1 in control *w*^*1118*^ (A), *Df* ^*rho-*1,3^ (B), *vn*^*L6*^ (C), and *Df* ^*rho-*1,3^ *vn*^*L6*^ double homozygotes at embryonic stage 13 (D). A mild increase in Dcp1 immunoreactivity is seen in *vn* and *rho*-1,3 single mutants (compared to *w*^*1118*^ controls). This signal is strongly enhanced in the double mutants. Scale bars 50 μm. Dcp1, Death caspase-1; *Df*, deficiency; *rho*-1, *rhomboid*; *rho*-3, *roughoid*; *vn*, *vein*; *w*^*1118*^, *white*^*1118*^.
(TIF)

S1 Table A genetic survey to identify regulators of apoptosis in patterning mutants. List of pathways targeted and transgenes used to assess the rescue of apoptosis in *ftz* mutants. In each instance, the transgene of interest was expressed globally using the *actin5C-*Gal4 driver and a positive score was attributed if a reduction in the levels of cleaved Dcp1 occurred. Dcp1, Death caspase-1; *ftz*, *fushi-tarazu*.
(XLSX)

S1 Movie *hid* is up-regulated at mid-embryogenesis in *ftz* mutants. Activity of the *hid*^*Δ*.*GFP*^ reporter in a *ftz*^*Δ*.*attP*^ mutant embryo. Negligible fluorescence is detected during the early stages of embryogenesis but around embryonic stage 11 (approximately 7 hours after egg laying) bands of fluorescence appear. Hours after egg lay are displayed in the lower right corner. *ftz*, *fushi-tarazu*; GFP, green fluorescent protein; *hid*, *head involution defective*.
(AVI)

S1 Data (XLSX)

## Abbreviations

A/P: anterior/posterior
APC: Adenomatous polyposis coli
Dcp1: Death caspase-1
Df: deficiency
dpERK: phosphorylated extracellular signal–regulated kinase
EGF: epidermal growth factor
EGFR: epidermal growth factor receptor
*ftz*: *fushi-tarazu*
GFP: green fluorescent protein
*hh*: *hedgehog*
*hid*: *head involution defective*
IAP: inhibitor of apoptosis protein
*kr*: *krüppel*
mSPIM: multiview single plane illumination microscopy
*odd*: *odd-skipped*
PBS: phosphate buffered saline
*ptc*: *patched*
*rpr*: *reaper*
*rho*-1: *rhomboid*
*rho*-3: *roughoid*
*skl*: *sickle*
TGF: transforming growth factor
*tll*: *tailless*
*vn*: *vein*
*wg*: *wingless*
