# Supplementary figures and images for: EGFR signaling coordinates patterning with cell survival during Drosophila epidermal development
Source: PLoS Biol. 2018 Oct 31;16(10):e3000027. doi: 10.1371/journal.pbio.3000027 (PMC6231689; doi:10.1371/journal.pbio.3000027)

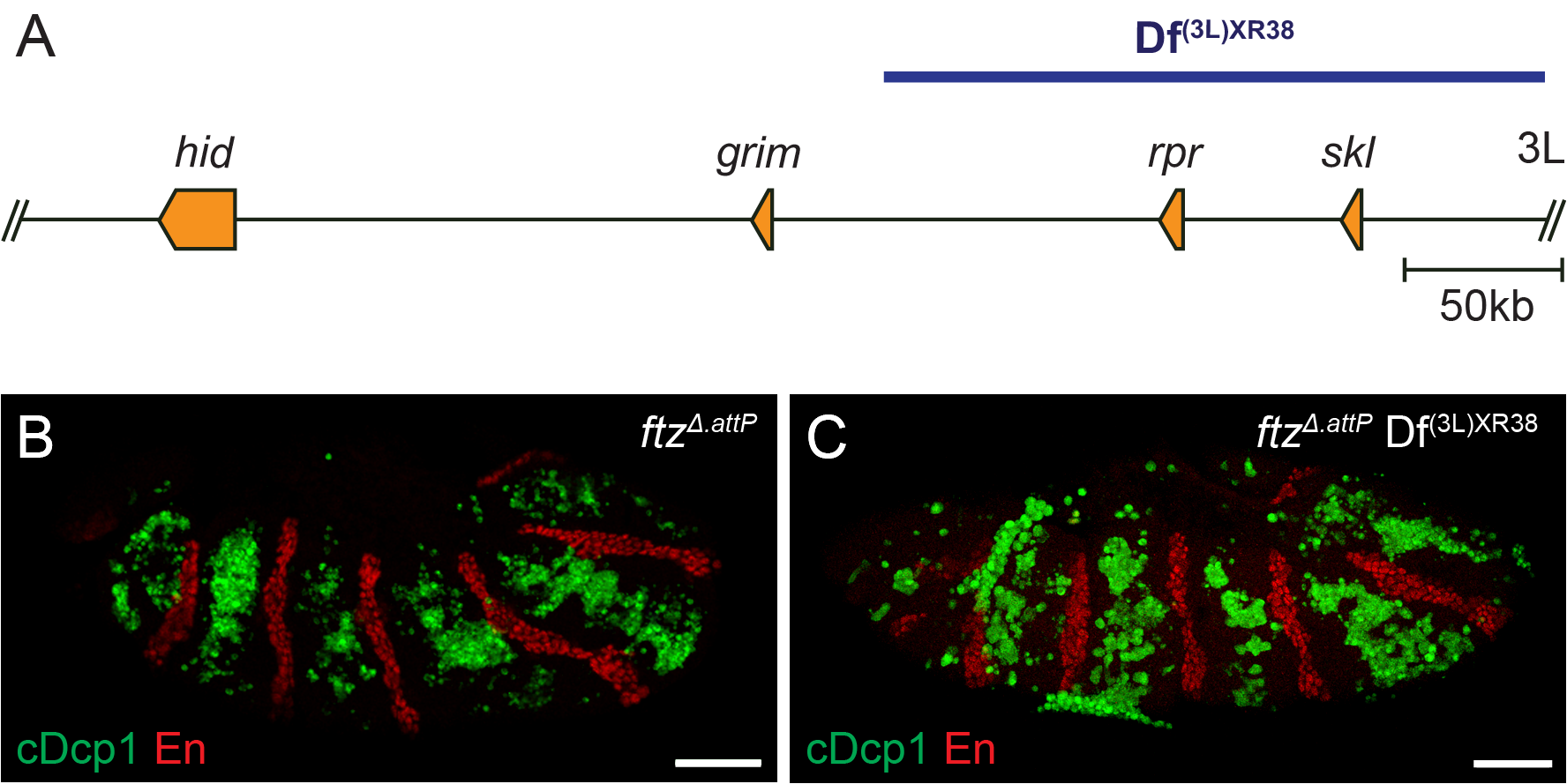

Supplement: S1 Fig — (A) Schematic representation of the Drosophila H99 locus showing the genes encoding the four major IAP antagonists. The Df(3L)XR38 deficiency, which removes the rpr and skl genes, is highlighted in blue. (B, C) Cleaved Dcp1 immunoreactivity in stage 12 ftzΔ.attP (B) and ftzΔ.attP Df(3L)XR38 homozygotes (C). Scale bars 50 μm. Df, deficiency; ftz, fushi-tarazu; IAP, inhibitor of apoptosis protein; rpr, reaper; skl, sickle. (TIF) [file pbio.3000027.s001.tif]

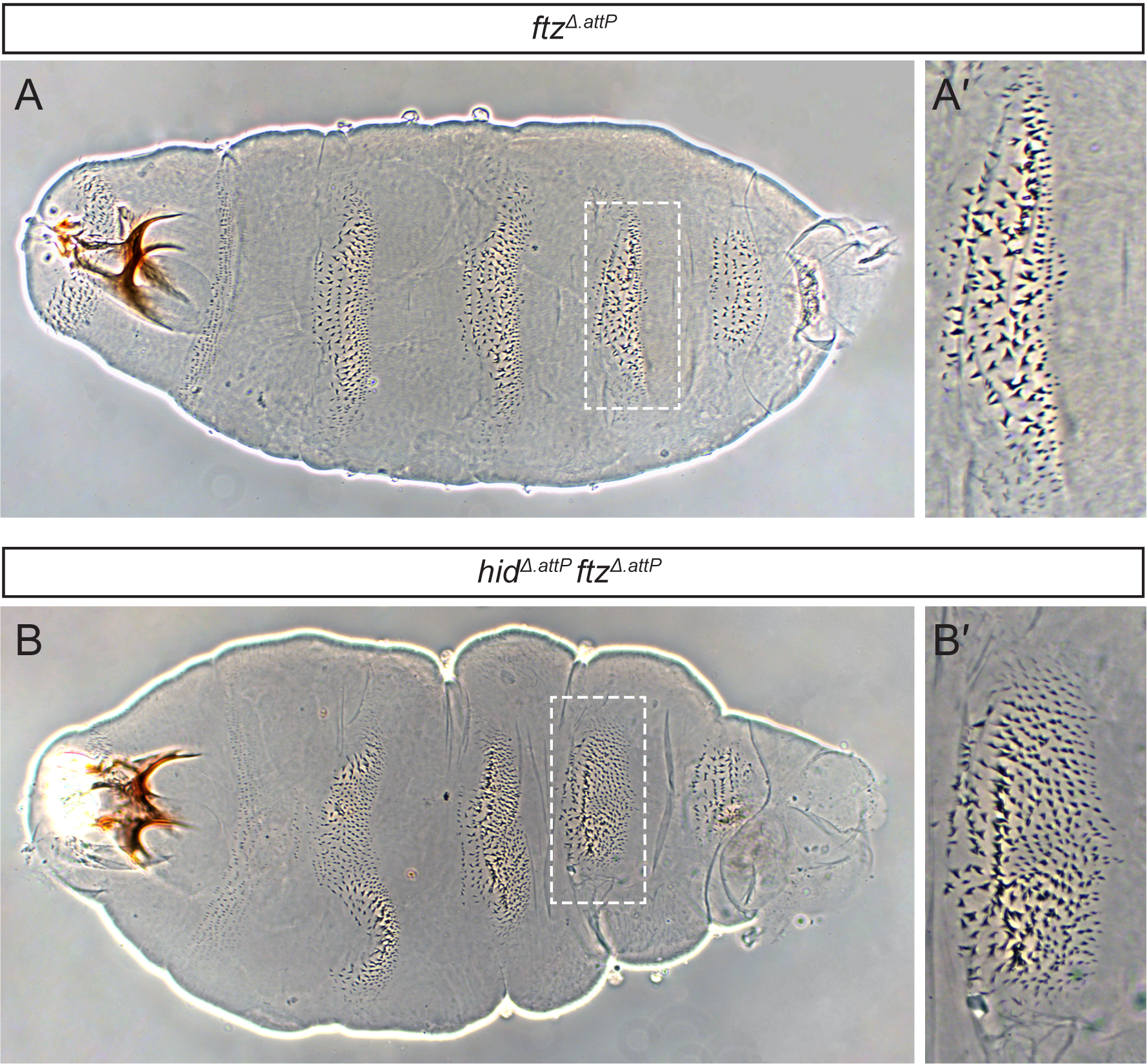

Supplement: S2 Fig — (A, B) Cuticle preparations of ftzΔ.attP (A) and hidΔ.attP ftzΔ.attP (B) embryos. In ftzΔ.attP homozygotes, remaining denticle belts have near wild-type morphology. In hidΔ.attP ftzΔ.attP homozygotes, the remaining denticle belts are expanded posteriorly with multiple rows of nondescript ectopic denticles (B′). ftz, fushi-tarazu; hid, head involution defective. (TIF) [file pbio.3000027.s002.tif]

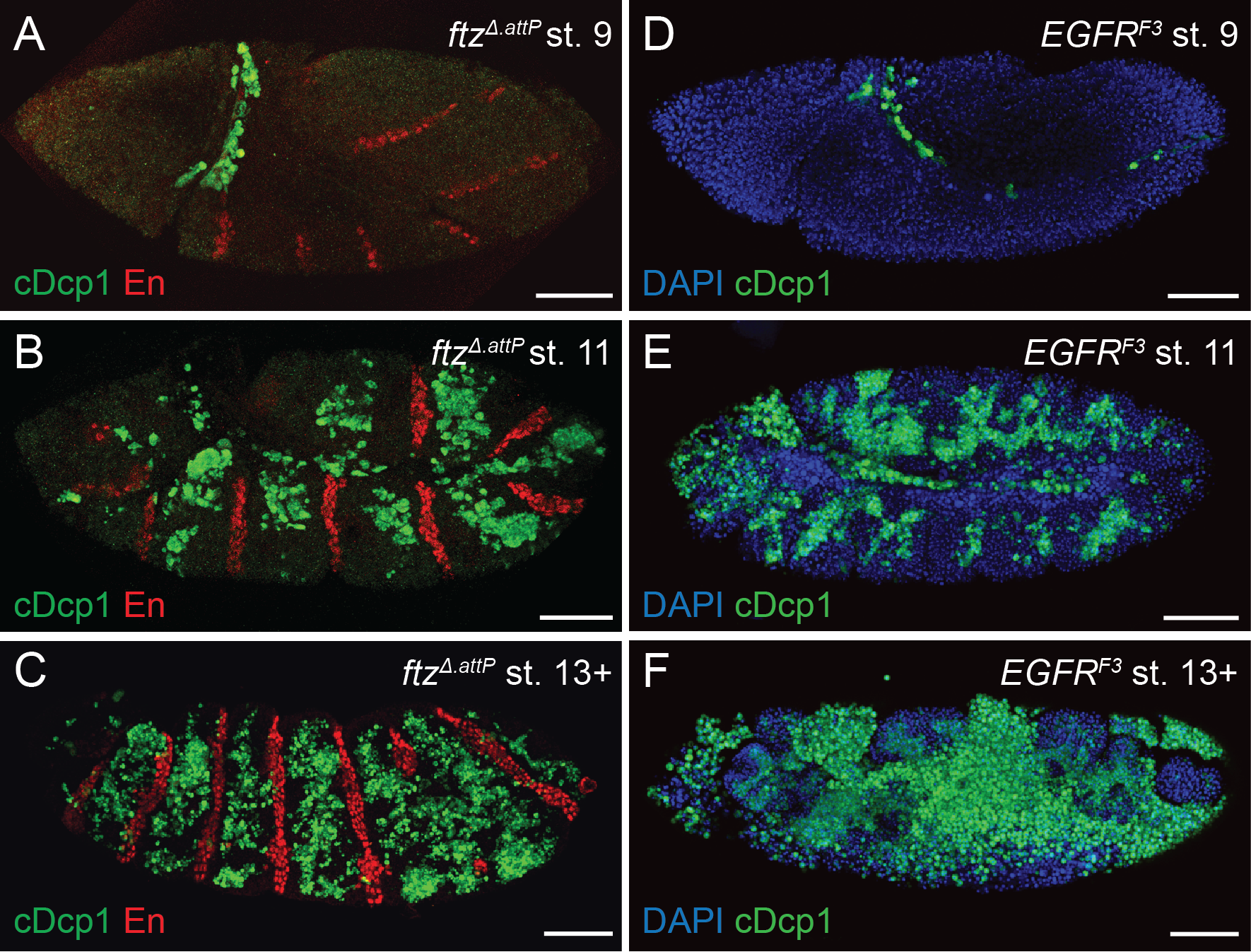

Supplement: S3 Fig — (A–C) Cleaved Dcp1 immunoreactivity in ftzΔ.attP mutants at embryonic stages 9 (A), 11 (B), and 13+ (C). (D–F) Cleaved Dcp1 immunoreactivity in EGFRF3 mutants at embryonic stages 9 (D), 11 (E), and 13+ (F). In both genotypes, cleaved Dcp1 is first detected in stage 11 and persists throughout the remainder of embryonic development. Scale bars 50 μm. Dcp1, Death caspase-1; EGFR, epidermal growth factor receptor; ftz, fushi-tarazu. (TIF) [file pbio.3000027.s003.tif]

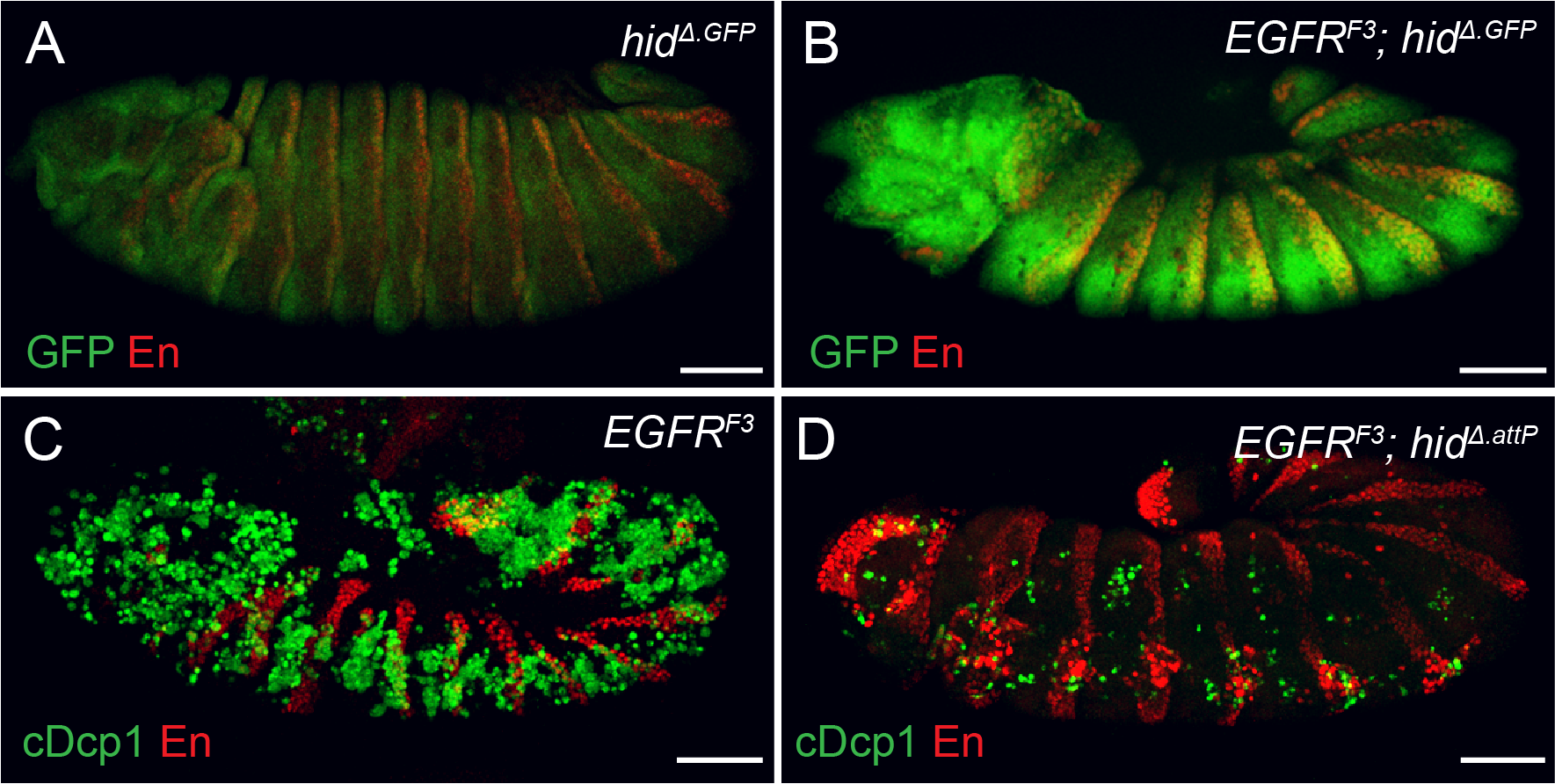

Supplement: S4 Fig — (A, B) Transcription of hid, as assayed with the hidΔ.GFP reporter, in control (A) and EGFRF3 mutant (B) stage 12 embryos. hid is up-regulated in most epidermal cells upon loss of EGFR signaling. (C, D) Cleaved Dcp1 immunoreactivity is strongly up-regulated throughout the epidermis in stage 12 EGFRF3 single mutants (C) and this up-regulation is lost in stage 12 EGFRF3; hidΔ.attP double homozygotes (D). Scale bars 50 μm. Dcp1, Death caspase-1; EGFR, epidermal growth factor receptor; GFP, green fluorescent protein; hid, head involution defective. (TIF) [file pbio.3000027.s004.tif]

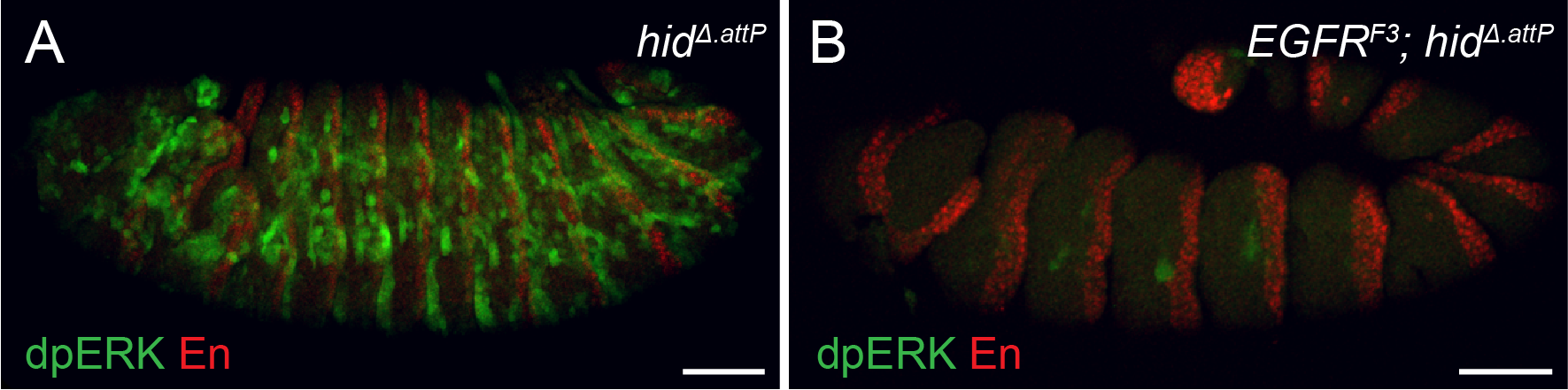

Supplement: S5 Fig — (A, B) dpERK immunoreactivity in control (EGFR+/+) hidΔ.attP (A) and EGFRF3; hidΔ.attP double homozygotes. Extensive dpERK immunoreactivity is detected in wild-type control embryos (A) and this signal is largely lost in EGFRF3; hidΔ.attP double mutants (B). We take this as evidence that EGFR signaling is the major source of ERK phosphorylation in the embryonic epidermis. Scale bars 50 μm. dpERK, phosphorylated extracellular signal–regulated kinase; EGFR, epidermal growth factor receptor; hid, head involution defective. (TIF) [file pbio.3000027.s005.tif]

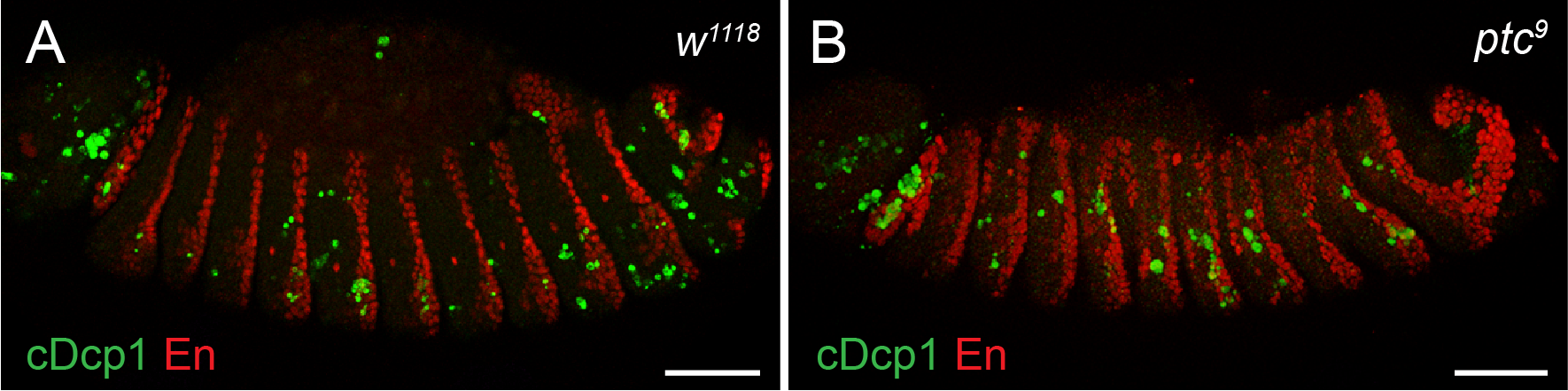

Supplement: S6 Fig — (A, B) Cleaved Dcp1 immunoreactivity in control w1118 (A) and ptc9 mutant embryos (B) at embryonic stage 13. No increase in Dcp1 cleavage was detected in ptc mutants, despite disruption to the segmental pattern. Scale bars 50 μm. Dcp1, Death caspase-1; ptc, patched; w1118, white1118. (TIF) [file pbio.3000027.s006.tif]

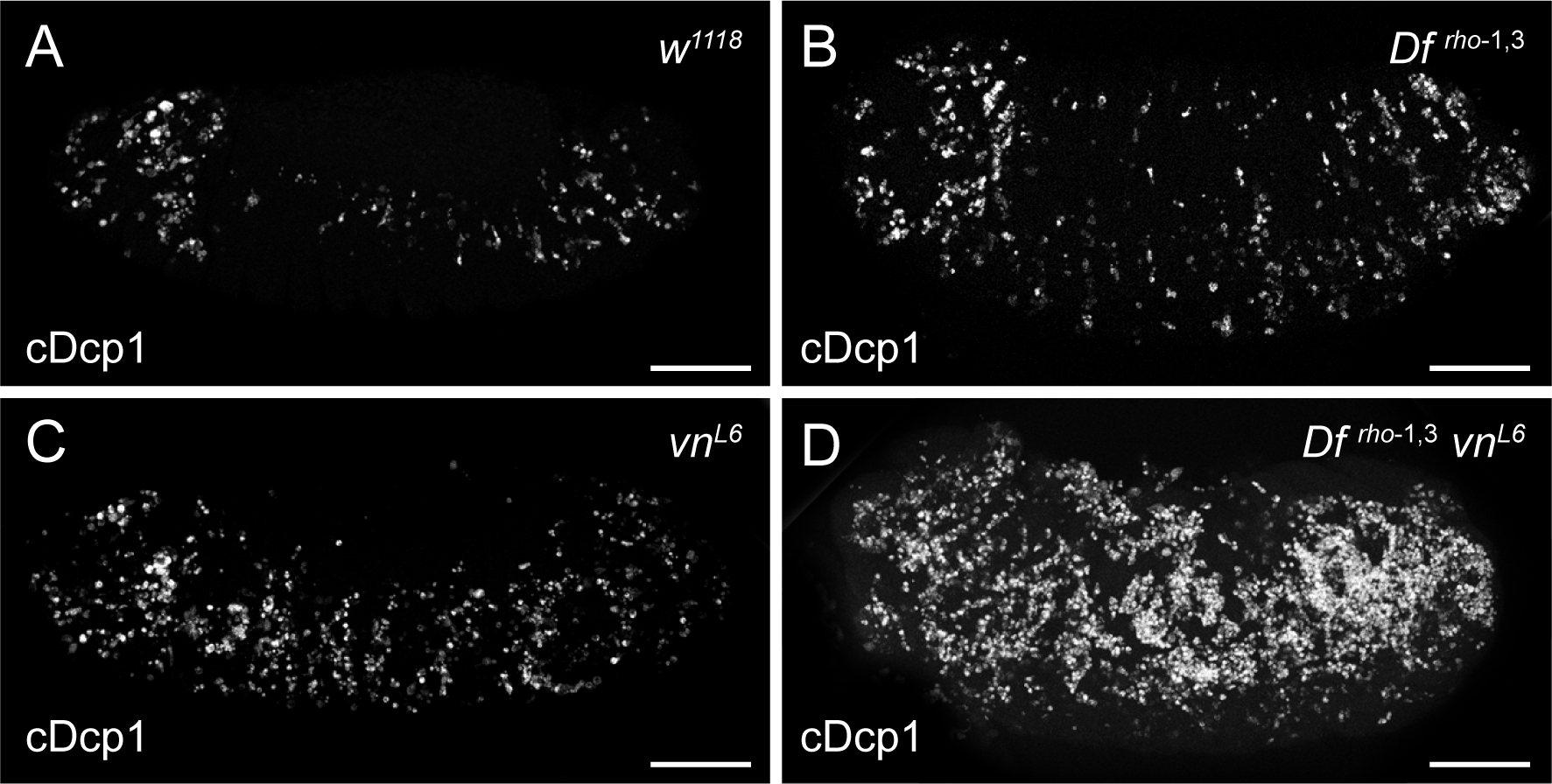

Supplement: S7 Fig — (A–D) Cleaved Dcp1 in control w1118 (A), Df rho-1,3 (B), vnL6 (C), and Df rho-1,3 vnL6 double homozygotes at embryonic stage 13 (D). A mild increase in Dcp1 immunoreactivity is seen in vn and rho-1,3 single mutants (compared to w1118 controls). This signal is strongly enhanced in the double mutants. Scale bars 50 μm. Dcp1, Death caspase-1; Df, deficiency; rho-1, rhomboid; rho-3, roughoid; vn, vein; w1118, white1118. (TIF) [file pbio.3000027.s007.tif]
